# PVA–Borax Hydrogels Loaded with Mono- and Bis-Spiro-Dioxy-Biphenyl-Cyclotriphosphazenes: Fabrication, Physicochemical Properties, and Release Kinetics

**DOI:** 10.3390/molecules31142463

**Published:** 2026-07-14

**Authors:** Seda Demirel Topel

**Affiliations:** Department of Electrical and Electronics Engineering, Faculty of Engineering and Natural Sciences, Antalya Bilim University, Dosemealti, 07190 Antalya, Türkiye; seda.demireltopel@antalya.edu.tr

**Keywords:** phosphazene, PVA, hydrogel, release kinetics

## Abstract

Spiro-dioxy-biphenyl cyclotriphosphazene derivatives, namely mono-spiro cyclotriphosphazene (SCP) and bis-spiro cyclotriphosphazene (Bis SCP), were incorporated into poly(vinyl alcohol) (PVA)–borax hydrogels to investigate the effect of phosphazene architecture on hydrogel properties and release behavior. Hydrogels containing 5 and 10 wt% phosphazene derivatives were prepared by borax crosslinking combined with freeze–thaw gelation and characterized by SEM, FTIR, thermogravimetric analysis, swelling measurements, rheological analysis, and release kinetics. SEM analysis revealed that phosphazene incorporation modified the hydrogel morphology and increased network heterogeneity. Swelling behavior strongly depended on phosphazene structure; 5 wt% SCP/PVA exhibited the highest equilibrium swelling ratio (~990%), whereas Bis SCP-containing hydrogels showed lower swelling capacities (~290–350%). Rheological measurements confirmed gel-like behavior (G′ > G″) for all formulations, and SCP-loaded hydrogels exhibited greater mechanical reinforcement than Bis SCP-loaded systems. Thermal analysis demonstrated improved thermal stability with increasing phosphazene content. Release studies performed in PBS/DMSO (1:1, pH = 7.4) revealed diffusion-controlled transport. The Higuchi (R^2^ = 0.994–0.995) and Korsmeyer–Peppas (R^2^ = 0.999) models provided the best fit, while diffusion exponent values (*n* = 0.365–0.396) indicated a mechanism of Fickian diffusion. These outcomes demonstrate that the degree of spiro substitution effectively governs the structure–property relationships of PVA–borax hydrogels for controlled-release applications.

## 1. Introduction

Poly(vinyl alcohol) (PVA)-based hydrogels have attracted significant scientific and technological interest over the past two decades owing to their exceptional biocompatibility, low cytotoxicity, high transparency, chemical resistance, film-forming ability, and the capacity to engage in both intermolecular hydrogen bonding and covalent or dynamic crosslinking [1]. PVA hydrogels can be fabricated through multiple strategies, including chemical crosslinking with glutaraldehyde or epichlorohydrin, radiation-induced crosslinking, and physical crosslinking via freeze–thaw cycling or ionic coordination, each conferring distinct mechanical signatures and network topologies [2]. Among physical crosslinking agents, sodium tetraborate decahydrate (borax) has attracted renewed attention as a remarkably simple yet effective gelator for PVA [3,4]. In aqueous solution, borax dissociates to yield the tetrahydroxyborate anion [B(OH)_4_]^−^, which undergoes rapid, reversible esterification with the 1,2-diol repeat units of PVA to form cyclic borate ester linkages [5]. These transient coordination crosslinks endow the resulting PVA–borax networks with self-healing behavior, shear-thinning flow, and pronounced stimulus responsiveness to pH and competing diol species [6]. For instance, Sun et al. prepared a multifunctional PVA/borate/ε-poly-L-lysine/hyaluronic acid hydrogel via a one-pot method, exhibiting ultrafast self-healing within 1 min, remarkable stretchability up to 15-fold its original length, moldability, and effective antibacterial activity against *S. aureus* and *E. coli*, making it a promising material for biomedical and soft robotic applications [7]. Moreover, the spatial density of crosslinks, and hence the elastic modulus, swelling degree, and effective mesh size of the network, can be precisely tuned by adjusting the PVA molecular weight, degree of hydrolysis, polymer concentration, and borax-to-PVA molar ratio [8]. Jiang et al. reported that increasing PVA concentration and decreasing crosslinker content enhanced hydrogel swelling, whereas higher crosslinker levels produced denser network structures; their numerical model successfully predicted the relationship between hydrogel microstructure and swelling behavior [9]. Ceylan et al. prepared borax-containing PVA/chitosan cryogels and demonstrated that borax-induced crosslinking improved mechanical strength, reduced swelling, and modified pore structure [10]. They also showed that chitosan molecular weight influenced boron release, highlighting the potential of these cryogels for bone tissue engineering applications. Due to their high water content, biocompatibility, tunable mechanical properties, and self-healing capability, PVA–borax hydrogels have attracted considerable attention for biomedical applications, particularly as wound dressings and wound-healing materials. Their ability to maintain a moist wound environment while serving as carriers for therapeutic agents makes them promising candidates for the development of advanced hydrogel-based wound care systems.

Functional hydrogels with tailored physicochemical profiles are increasingly achieved by incorporating molecular- or nanoscale additives that modulate network topology and introduce new stimulus-specific functionalities [11]. In this context, cyclotriphosphazene (CTP) derivatives have emerged as a uniquely powerful class of hybrid inorganic–organic building blocks [12]. The CTP scaffold consists of a fully delocalized six-membered N_3_P_3_ ring in which three phosphorus atoms alternate with three nitrogen atoms, with each phosphorus bearing two substituents that can be varied almost without restriction through nucleophilic substitution of the parent hexachlorocyclotriphosphazene (HCCP) [13]. This exceptional structural versatility, together with the high thermal stability (typically exceeding 300 °C), low dielectric constant, and diverse biological activities of the phosphazene ring, has attracted increasing interest in the use of CTP derivatives as functional components in biomaterials, particularly for drug delivery, wound healing, and wound dressing applications [14]. Importantly, the nitrogen and oxygen atoms associated with the N_3_P_3_ ring present Lewis basic sites and hydrogen bond acceptors capable of interacting with hydroxyl-rich polymer backbones through hydrogen bonding, while the electrophilic phosphorus centers bearing chloro substituents may participate in coordination-type interactions, properties of direct relevance to their integration into PVA matrices and their potential to modulate network crosslink density [15]. Among the many substitution patterns accessible on the N_3_P_3_ scaffold, spiro-dioxy substitution with bidentate aromatic diol ligands represents an architecturally distinctive and synthetically well-controlled strategy [16]. When 2,2′-dihydroxybiphenyl (biphenol) reacts with HCCP under mild basic conditions, the two phenolic oxygen atoms chelate adjacent phosphorus substituent positions in a spiro fashion, generating a rigidly fused bicyclic motif appended to the central N_3_P_3_ ring. Depending on stoichiometry and reaction temperature, mono-spiro (SCP, retaining four P–Cl bonds), bis-spiro (Bis SCP, retaining two P–Cl bonds), and tris-spiro (Tri SCP, fully substituted) products are obtained with high regioselectivity and well-defined molecular identity [17]. The progressive replacement of reactive P–Cl bonds by spiro-biphenyldioxy units systematically modifies molecular symmetry, free volume, conformational rigidity, and intermolecular interaction capacity. SCP presents a larger, more Lewis-acidic molecular surface with four residual chlorine atoms capable of accepting hydrogen bonds, whereas Bis SCP adopts a bulkier, more sterically encumbered architecture with only two residual chlorines and a markedly different spatial footprint [17,18]. These contrasting structural features are anticipated to translate into distinct modes of interaction with the PVA–borax network, influencing polymer chain mobility, water accessibility, and ultimately the transport kinetics of the phosphazene guest. Owing to their chemical interactions with PVA, these materials have been investigated for applications in wound dressings, rapid hemostatic sealing, and the repair of infected wounds. Ni et al. synthesized a phosphazene-based PVA–borax hydrogel for an adhesive hydrogel that exhibited a 4 h antibacterial property, which also shortened the bleeding by 88%, and accelerated wound healing [19]. Zhang et al. developed a barnacle-inspired polyphosphazene–PVA hydrogel (PZBA–PVA) as an injectable antibacterial wound dressing, exhibiting strong antimicrobial activity, good biocompatibility, and enhanced healing of infected wounds through hemostatic and anti-inflammatory effects [20]. Additionally, Ozay et al. employed hexakis(4-formylphenoxy)cyclotriphosphazene as a novel crosslinking agent, leveraging its six reactive functional groups to form effective bridges between polymeric chains [21]. The resulting hydrogel was subsequently loaded with Amoxicillin, a well-established bacteriostatic agent, and its antibacterial performance was evaluated. The same research team synthesized hexakis [4-(acrylamido)phenoxy]cyclotriphosphazene (HAAP), a phosphazene-based cross-linker, in a follow-up study. This cross-linker was employed to construct thermo- and pH-sensitive p(NIPAM-co-VI) hydrogels, showcasing the adaptability of cyclotriphosphazene derivatives in adjusting stimuli-responsive behavior [22]. More recently, Yudaev et al. developed a silver-loaded hydrogel based on an aryloxycyclotriphosphazene and polyvinyl alcohol bearing both *p*-formylphenoxy and *β*-carboxyethenylphenoxy pendant groups [23]. In vivo assessment of the wound-healing potential of this formulation revealed a statistically significant reduction in wound area of 91.43% (*p* < 0.05) in the rabbit hind limb model by the tenth day of observation, highlighting the promising therapeutic efficacy of phosphazene-based hydrogel systems.

Although phosphazene-based hydrogels and phosphazene-containing PVA composites have been reported in the literature, these systems have predominantly relied on covalently incorporated polyphosphazene structures, phosphazene-based crosslinkers, or inorganic phosphorus-containing additives. In contrast, the incorporation of well-defined small-molecule spiro-dioxy-biphenyl cyclotriphosphazene derivatives into PVA–borax hydrogels has not been systematically investigated. Consequently, the influence of the degree of spiro substitution on network structure, viscoelastic behavior, swelling characteristics, and release kinetics remains unclear. Addressing this knowledge gap is important for understanding physicochemical relationships and evaluating the potential of spiro-cyclotriphosphazene derivatives as functional additives in PVA-based hydrogels for biomedical applications, including wound healing and wound dressing.

Accordingly, two spiro-dioxy-biphenyl cyclotriphosphazene derivatives, mono-spiro tetrachloro compound (SCP) and bis-spiro dichloro compound (Bis SCP), were synthesized from HCCP and 2,2′-dihydroxybiphenyl and incorporated into PVA–borax hydrogels via a combined borax-crosslinking and freeze–thaw gelation protocol at two loading levels (5 and 10 wt%). The resulting hybrid hydrogels were comprehensively characterized by Fourier-transform infrared (FTIR) spectroscopy, scanning electron microscopy (SEM), thermogravimetric analysis (TGA), and oscillatory rheology to elucidate the effects of phosphazene structure on intermolecular interactions, morphology, thermal stability, and viscoelastic behavior. In addition, phosphazene release in PBS (1:1, pH 7.4) was monitored by UV–Vis spectroscopy and analyzed using zero-order, first-order, Higuchi, Hixson–Crowell, and Korsmeyer–Peppas kinetic models, with apparent diffusion coefficients estimated from Fickian diffusion theory. Through this approach, the present study establishes structure–property–release relationships for spiro-cyclotriphosphazene-functionalized PVA hydrogels and evaluates the effect of the degree of spiro substitution on their functional performance.

## 2. Results and Discussion

### 2.1. Synthesis and Characterization of SCP and Bis SCP Compounds

SCP and Bis-SCP were synthesized via nucleophilic substitution reactions of hexachlorocyclotriphosphazene (HCCP) with 2,2′-biphenol in the presence of K_2_CO_3_ as base, under an inert argon atmosphere [24]. The reaction outcomes were highly dependent on the stoichiometric ratio of biphenol to HCCP, thereby enabling selective access to either mono- or di-substituted phosphazene products. SCP was obtained in 90% yield by reacting HCCP with one equivalent of 2,2′-biphenol (1:1 molar ratio), affording a single spiro-substituted product in which one geminal chlorine pair on the phosphazene ring is replaced by a bidentate biphenolate unit (Figure S1). Bis-SCP was synthesized under analogous conditions but employing an approximately 2:1 molar ratio of 2,2′-biphenol to HCCP, promoting double spiro-substitution at two geminal phosphorus centers (Figure S2). Notably, the reaction required a significantly extended reaction time of 2 h compared to the 15 min sufficient for SCP, consistent with the reduced electrophilicity of the phosphazene ring following the first substitution event. In the ^1^H-NMR spectrum of SCP, the signals corresponding to the aromatic ring protons were observed at 7.33–7.61 ppm (m, 8H, C_12_H_8_), whereas Bis-SCP exhibited aromatic proton signals at 7.35–7.55 ppm (m, 16H, C_12_H_8_). Furthermore, the absence of any signal attributable to hydroxyl (–OH) protons confirms the successful formation of the target structures, indicating the complete consumption of the hydroxyl groups during the substitution reaction (Figures S4 and S8). The ^13^C-NMR spectra of SCP and Bis-SCP exhibited the characteristic aromatic carbon resonances in the range of 121.63–148.56 ppm, corresponding to the assigned carbon atoms of the aromatic ring. (Supporting Information, Figures S5 and S9). The ^31^P-NMR spectrum of SCP exhibited two characteristic phosphorus resonances at δ 12.84 ppm (*t*, 1P) and 24.52 ppm (*d*, 2P). Similarly, the ^31^P-NMR spectrum of Bis-SCP showed signals at δ 19.90 ppm (*d*, 2P, P(O_2_C_12_H_8_)) and 29.18 ppm (*t*, 1P, PCl_2_). The presence of only the expected phosphorus resonances and the absence of additional signals confirm the successful synthesis and high purity of both compounds (Supporting Information, Figures S6 and S10).

### 2.2. Production of SCP/PVA and Bis SCP/PVA Hydrogels

**Figure 1:**
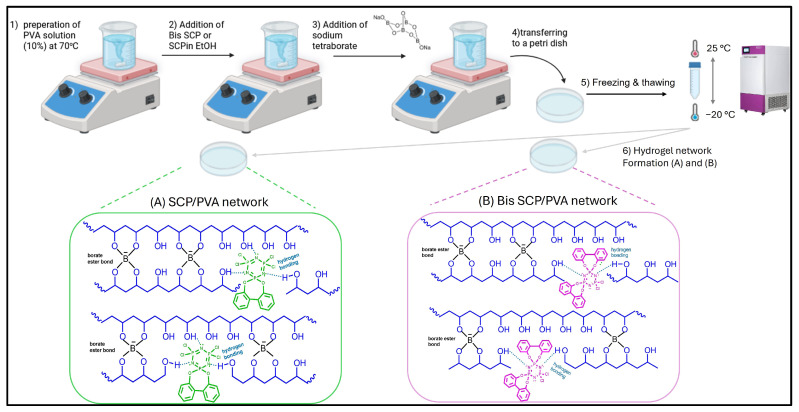
Schematic representation of the fabrication of (**A**) SCP/PVA, (**B**) Bis SCP/PVA hydrogels and their proposed crosslinked network structures.

Poly(vinyl alcohol) (PVA)-based hydrogels incorporating SCP and Bis-SCP were prepared by physical crosslinking with sodium tetraborate, a well-established method for producing dynamic, reversible hydrogel networks (Figure 1). In this approach, the tetraborate anion acts as a non-covalent difunctional crosslinker, forming cyclic boronate ester complexes with the *cis*-diol units of the PVA chains, thereby generating a three-dimensional polymeric network capable of retaining large amounts of water [25]. The two compounds differ in their degree of spiro-substitution and residual chlorine content: SCP retains four P–Cl bonds alongside a single biphenolate bridge, while Bis-SCP bears two biphenolate units and only two remaining chlorine atoms. This difference in chemical architecture is expected to influence the compatibility and interactions of each additive with the PVA matrix. The greater number of reactive P–Cl bonds retained in SCP may facilitate secondary interactions with the hydroxyl groups of the PVA chains, potentially promoting stronger interfacial compatibility within the hydrophilic network, while the more symmetric and less polar Bis-SCP, bearing a higher organic character due to its two biphenolate units, may achieve more homogeneous dispersion throughout the PVA matrix.

### 2.3. Morphology and Elemental Composition of the SCP and Bis SCP/PVA Hydrogels

**Figure 2:**
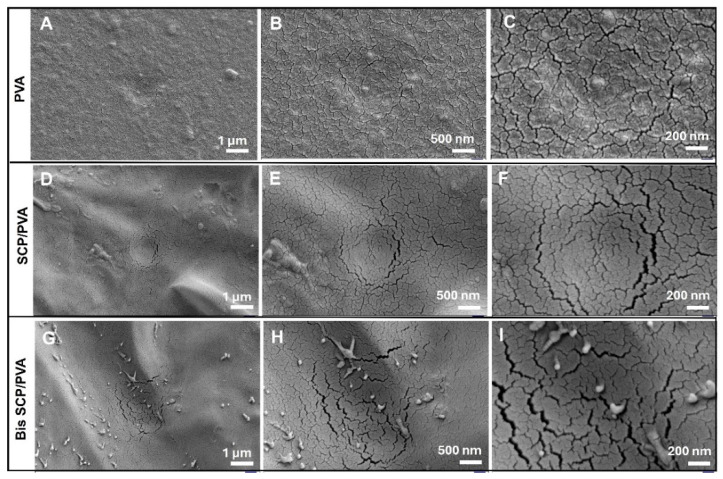
SEM micrographs showing the surface morphology of PVA, SCP/PVA, and Bis SCP/PVA hydrogels at different magnifications. (**A**–**C**) Neat PVA hydrogel, (**D**–**F**) SCP/PVA hydrogel, and (**G**–**I**) Bis SCP/PVA hydrogel. Images were acquired at progressively higher magnifications, with scale bars of 1 μm (**A**,**D**,**G**), 500 nm (**B**,**E**,**H**), and 200 nm (**C**,**F**,**I**).

The surface morphology of PVA–borax hydrogels and elemental compositions of phosphazene-loaded formulations were investigated by SEM-EDS (Figure 2 and Figures S11–S13). Pristine PVA hydrogel (Figure 2A–C) exhibited a relatively homogeneous surface with fine, interconnected domains and shallow cracking, consistent with formation of a physically crosslinked PVA–borax network; EDS confirmed the expected C/N/O composition with no detectable phosphorus or chlorine (Figure S11). Incorporation of SCP (Figure 2D–F) produced a modestly rougher surface with a more visible crack network, while Bis SCP-loaded hydrogels (Figure 2G–I) showed more pronounced surface roughness and wider, more extensively interconnected cracks. As these samples were imaged in the dried state under high-vacuum conditions, the observed features are influenced by drying-induced shrinkage. SEM-EDS confirmed successful incorporation of both derivatives, with comparable phosphorus content (~28–29 wt%) and markedly higher chlorine content in SCP/PVA (36.59 wt%) than in Bis SCP/PVA (12.77 wt%), consistent with the greater number of residual P–Cl substituents in SCP.

### 2.4. FTIR

**Figure 3:**
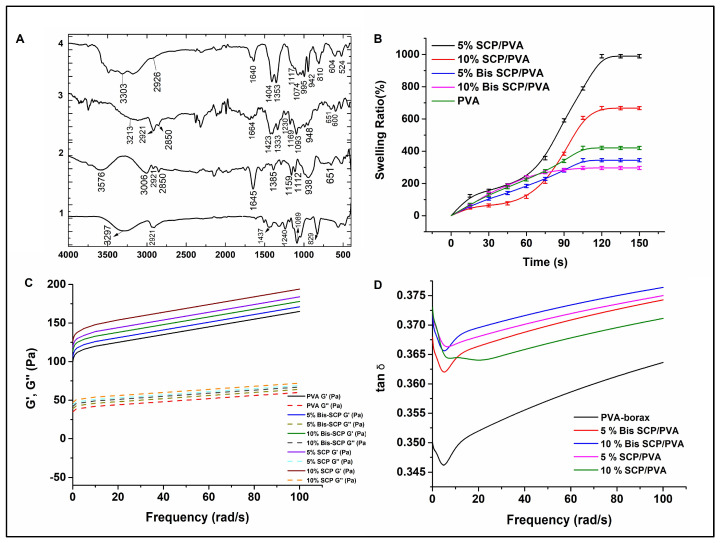
(**A**) FTIR spectra of neat PVA (**1**), PVA/borax hydrogel (**2**), Bis SCP/PVA hydrogel (**3**), and SCP/PVA hydrogel (**4**), showing the characteristic functional groups and interactions after hydrogel formation. (**B**) Swelling ratio of PVA, SCP/PVA, and Bis SCP/PVA hydrogels with different SCP or Bis SCP contents (5 and 10 wt%) (mean ± SD, *n* = 3). (**C**) Frequency sweep measurements showing the storage modulus (G′, solid lines) and loss modulus (G″, dashed lines) of the hydrogels over the investigated angular frequency range. (**D**) Frequency dependence of the loss factor (tan δ = G″/G′) for the corresponding hydrogels.

FTIR spectroscopy was utilized to investigate the chemical structures of the phosphazene derivatives and to evaluate their interactions with the PVA hydrogel network. (Figure 3A). The spectrum of neat PVA (Figure 3A-1) displayed the characteristic broad absorption centered at 3297 cm^−1^, attributable to O–H stretching associated with intermolecular hydrogen bonding, together with stretching of aliphatic C–H at 2921 cm^−1^ and C–O at 1240 and 1089 cm^−1^. Following hydrogel formation (Figure 3A-2), the stretching vibrations of the O–H group shifted to 3576 cm^−1^ and broadened, reflecting changes in hydrogen-bonding interactions induced by water absorption and crosslinking. The bands at 2921 and 2850 cm^−1^ correspond to C–H stretching vibrations. The peak at 1645 cm^−1^ is attributed to the bending vibration of absorbed H_2_O molecules. The band at 1385 cm^−1^ confirms borate ester formation between borate ions and PVA, while the peak at 1159 cm^−1^ is assigned to C–O stretching. The appearance of a band at 651 cm^−1^ corresponds to B–O–B vibrations, further supporting borate crosslink formation within the hydrogel network.

In the FTIR spectra of Bis-SCP, a broad band around 3213 cm^−1^ was assigned to stretching of O–H vibrations of hydroxyl groups and absorbed water, while the peaks at 2921 and 2850 cm^−1^ corresponded to C–H stretching vibrations. The band at 1664 cm^−1^ was attributed to the bending vibration of adsorbed water molecules. Aromatic C=C stretching vibrations appeared at 1423 and 1333 cm^−1^. The characteristic stretching of P=N groups in the phosphazene ring was observed at 1230, 1169, and 1093 cm^−1^, whereas the stretching of P–O–C vibrations was detected at 948 cm^−1^. The B–O–B bending vibration was identified at 651 cm^−1^.

For SCP, a broad O–H stretching band was observed around 3303 cm^−1^, and the bending vibration of adsorbed water appeared at 1640 cm^−1^. The aromatic C=C stretching vibrations were located at 1408 and 1353 cm^−1^. The characteristic stretching bands for the P=N group were observed at 1117, 1074, and 995 cm^−1^, while the P–O–P stretching vibration appeared at 942 cm^−1^. The B–O–B bending vibration was detected at 604 cm^−1^. The FTIR spectra of the pure SCP and Bis-SCP compounds are provided in the Supporting Information (Figures S3 and S7). The changes in the FTIR characteristics are summarized in Table 1, with notable shifts in the characteristic phosphazene ring vibrations after incorporation into the PVA/borax hydrogel.

The decrease in the *ν(P=N)* and *ν(P–O–C)* wavenumbers (cm^−1^) after incorporation into the PVA/borax hydrogel indicates a change in the chemical environment of the phosphazene moieties (Table 1). These observations suggest that the SCP is more favorable for intermolecular interactions with PVA than that of Bis SCP. The lower steric hindrance around the phosphazene ring may increase the accessibility of the ring nitrogen atoms for hydrogen-bond formation with PVA hydroxyl groups, while the residual P–Cl bonds may contribute additional secondary polar interactions. These interactions may weaken or elongate the P=N and P–O–C bonds, resulting in a redshift to lower wavenumbers, as observed in the reported studies [26,27,28].

### 2.5. Swelling Characteristics

The short-time swelling behavior of the hydrogel formulations was evaluated in deionized water over 150 s (Figure 3B). All formulations exhibited a rapid initial water uptake stage, followed by an apparent plateau within the investigated time window. The hydrogel swelling may continue over longer periods before reaching an osmotic equilibrium; the values obtained at 150 s are hereafter referred to as apparent swelling ratios. Pristine PVA hydrogel exhibited a swelling ratio of approximately 420% after 105–120 s, reflecting the hydrophilic nature of the PVA–borax network. The incorporation of phosphazene derivatives significantly affected the short-time swelling behavior. Among all formulations, 5% SCP/PVA showed the highest swelling ratio (~990% at 120 s), which may increase local free volume and enhance water uptake within the network. Increasing the SCP content to 10 wt% reduced the swelling ratio to approximately 670%, possibly due to partial network densification at higher additive concentrations.

In contrast, Bis SCP-containing hydrogels exhibited lower swelling ratios, reaching approximately 350% and 290% for 5% and 10% Bis SCP/PVA, respectively. This behavior is attributed to the bulkier, more rigid structure of Bis SCP, which may restrict polymer chain mobility and hinder water diffusion within the hydrogel matrix. This behavior parallels trends reported for other composite hydrogels, in which moderate incorporation of a functional additive enhances water uptake by increasing local free volume or introducing additional hydrophilic sites, before higher loadings begin to restrict swelling through network densification. For example, Song et al. developed a poly(acrylic acid-co-acrylamide) hydrogel reinforced with phytic acid (PA) and carboxymethyl cellulose (CMC) for ionic-conductive sensing applications, in which the unmodified PAA/AM network exhibited a swelling ratio of approximately 200% in deionized water, increasing to ~490% and ~450% upon the individual addition of PA or CMC, respectively, due to the hydrophilic and hydrogen-bond-forming character of each component [29]. Cheng et al. reported that the incorporation of graphene oxide into CMC/PVA/gelatin hydrogels increased equilibrium swelling to as much as 2139.73% by introducing additional hydroxyl groups capable of hydrogen bonding with water [30], while Abdelhaq et al. observed the opposite trend upon ZnO doping of alginate/acrylamide hydrogels, where increasing ZnO content from 5 to 15% progressively reduced swelling from 962% to 790% as the nanoparticles occupied pore volume within the network [31]. A similar reduction was reported by Gao et al., who found that increasing chitosan content in PAA/chitosan double-network hydrogels from 1% to 5% decreased the swelling ratio from 1774% to 1393% due to increased hydrogen-bond-mediated crosslinking density [32]. The present system reflects a comparable balance: while 5 wt% SCP promoted swelling, increasing the loading to 10 wt% reduced the ratio to approximately 670%, and the bulkier Bis SCP derivative produced substantially lower swelling values (approximately 350% and 290% for 5 and 10 wt%, respectively), consistent with restricted chain mobility arising from its more sterically demanding, doubly spiro-substituted architecture.

### 2.6. Viscoelastic Properties of SCP- and Bis SCP-Loaded Hydrogels

The viscoelastic behavior of pristine PVA–borax and phosphazene-loaded hydrogels was evaluated by oscillatory frequency sweep measurements through determination of storage modulus (G′), loss modulus (G″), and damping factor (tanδ) (Figure 3C,D). As shown in Figure 3C, all formulations exhibited G′ values higher than G″ throughout the investigated frequency range, indicating dominant elastic behavior and successful hydrogel formation. No crossover between G′ and G″ was observed, confirming preservation of solid-like network characteristics. The storage modulus increased after incorporation of phosphazene derivatives and followed the order: 10%  SCP/PVA>5%  SCP/PVA>10%  Bis-SCP/PVA>5%  Bis-SCP/PVA>PV *A hydrogel*.

Among all formulations, SCP-PVA hydrogels exhibited the highest G′ values, indicating more effective reinforcement of the PVA–borax network than Bis SCP/PVA. Although Bis SCP contains two spiro(2′,2″-dioxy-1′,1″-biphenylyl) groups, the increased steric bulk may reduce efficient chain organization within the hydrogel matrix. The calculated tanδ values remained below 1 for all samples (Figure 3D), indicating that elastic response remained dominant over viscous dissipation. Only minor variations in tanδ were observed, suggesting that phosphazene incorporation mainly affected network stiffness while preserving the overall viscoelastic nature of the hydrogels.

### 2.7. Thermal Stability of SCP- and Bis SCP-Loaded PVA Hydrogels

**Figure 4:**
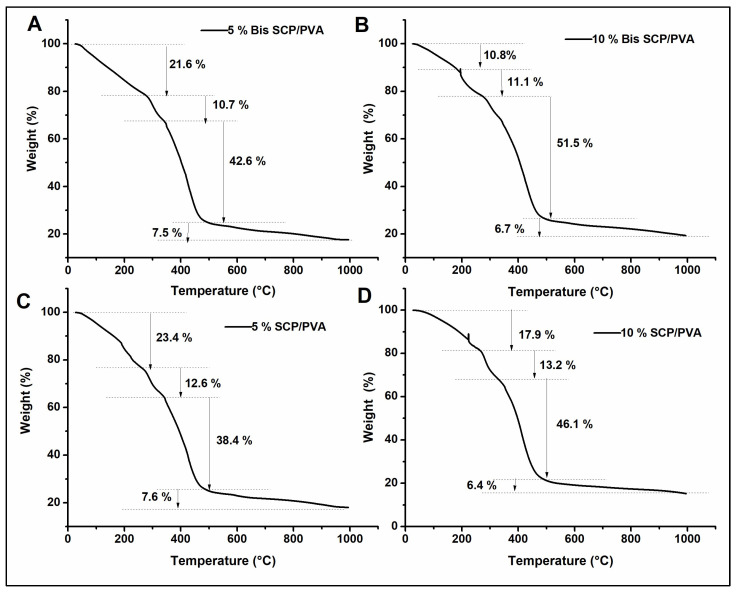
TG curves of PVA hydrogels containing different amounts of SCP and Bis SCP: (**A**) 5 wt% Bis SCP/PVA, (**B**) 10 wt% Bis SCP/PVA, (**C**) 5 wt% SCP/PVA, and (**D**) 10 wt% SCP/PVA.

The thermal behavior of phosphazene-loaded PVA hydrogels was investigated by TG analysis, and the corresponding degradation profiles are presented in Figure 4. All formulations exhibited a multistep degradation pattern, indicating sequential removal of physically associated species and decomposition of the polymer network. The initial weight loss below approximately 250 °C was attributed to the evaporation of adsorbed and bound water, as well as the elimination of residual hydroxyl-containing species. In this region, weight losses of 21.6% and 10.8% were observed for 5% and 10% Bis SCP/PVA, respectively, whereas 5% and 10% SCP/PVA showed losses of 23.4% and 17.9%, indicating reduced low-temperature mass loss at higher phosphazene loading. The second degradation stage, occurring between approximately 250 and 400 °C, is attributed to the partial cleavage of PVA chains and the decomposition of borate-associated network structures. In this interval, 10% Bis SCP/PVA (11.1%) and 10% SCP/PVA (13.2%) exhibited slightly higher mass loss than their corresponding 5% formulations (10.7% and 12.6% for Bis SCP/PVA and SCP/PVA, respectively). The major degradation step occurred within approximately 350–500 °C, corresponding to backbone degradation and carbonization of the hydrogel matrix. The largest mass loss was observed in this region, reaching 51.5% and 46.1% for 10% Bis SCP/PVA and 10% SCP/PVA, respectively, compared with 42.6% and 38.4% for the corresponding 5% systems. Notably, both 10% formulations exhibited higher residual mass at elevated temperatures, indicating improved thermal resistance. At temperatures approaching 1000 °C, all hydrogels retained measurable char residues (~15–20%), reflecting formation of thermally stable phosphorus-containing structures. The final weight-loss stage (500–1000 °C), accounting for approximately 6.4–7.6% of the total mass, is attributed to the gradual decomposition of the carbonaceous char. Among the investigated samples, 10% Bis-SCP/PVA exhibited the highest char residue at 1000 °C, indicating enhanced thermal stability compared to the SCP-containing hydrogels.

### 2.8. Release Kinetics of SCP- and Bis SCP from PVA Hydrogels

**Figure 5:**
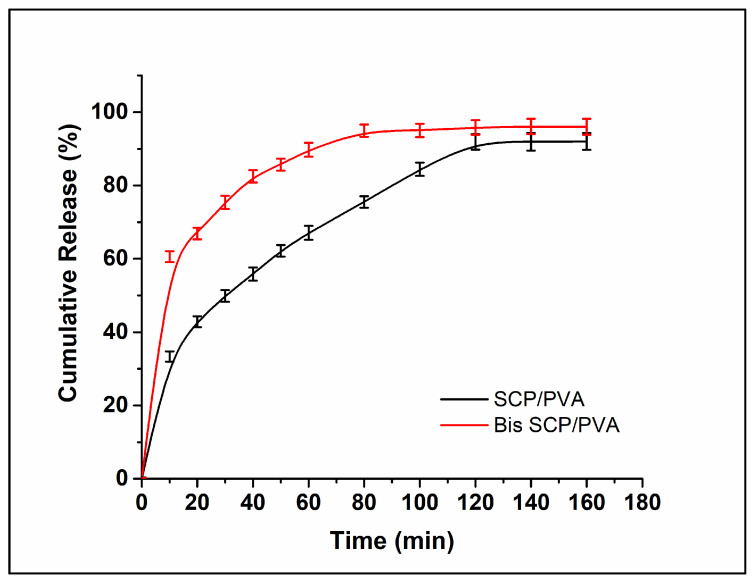
In vitro cumulative release profiles of SCP and Bis SCP from PVA hydrogels in phosphate-buffered saline (DMSO:PBS (1:1), pH 7.4) at 37 °C. Error bars represent the standard deviation of three independent measurements (mean ± SD, *n* = 3).

Prior to kinetic modeling, calibration curves for 10% SCP and 10% Bis SCP were established in a DMSO (1:1, *v*/*v*) medium to enable accurate quantification of phosphazene release. The corresponding calibration plots are presented in Figure S14. Subsequently, time-dependent release studies were conducted over 160 min for both compounds (Figure S15), and the cumulative release percentages vs. time are shown in Figure 5. SCP/PVA exhibited a gradual release behavior, reaching approximately 92% release within 160 min. In contrast, Bis SCP/PVA showed a faster initial release, reaching approximately 96% within the same period. For both formulations, the release curves approached a plateau after approximately 120 min, indicating that most of the embedded SCP had been released by this time. Release experiments were performed in triplicate (*n* = 3), and the mean cumulative release values were used for kinetic modeling.

The cumulative release profiles of SCP and Bis SCP from their respective PVA–borax hydrogel matrices were analyzed using five mathematical models, namely zero-order, first-order, Hixson–Crowell, Higuchi, and Korsmeyer–Peppas models, to identify the dominant transport mechanism governing phosphazene release (Table 2).

**Figure 6:**
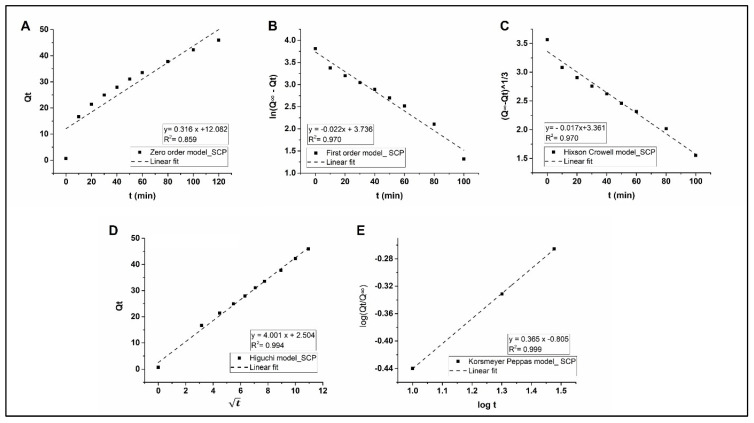
Linear fitting of the experimental release data for SCP released from PVA–borax hydrogels using different kinetic models: (**A**) zero-order, (**B**) first-order, (**C**) Hixson–Crowell, (**D**) Higuchi, and (**E**) Korsmeyer–Peppas.

For the SCP/PVA hydrogel (Figure 6), the zero-order model (Figure 6A) exhibited the lowest correlation coefficient (R^2^ = 0.859), indicating that the release did not occur at a constant rate over time. This behavior suggests that the driving force for release progressively decreases as the concentration gradient between the hydrogel matrix and the release medium diminishes. Improved fitting was obtained with the first-order model (Figure 6B, R^2^ = 0.970), indicating partial concentration dependence of the release process. Similarly, the Hixson–Crowell model (Figure 6C, R^2^ = 0.970) provided a comparable fit, suggesting that changes in the hydrogel matrix’s effective diffusion surface may contribute to the release behavior. The highest fitting accuracy was obtained using the Higuchi (Figure 6D, R^2^ = 0.994) and Korsmeyer–Peppas (Figure 6E, R^2^ = 0.999) models. The excellent agreement with the Higuchi model demonstrates that release was predominantly governed by diffusion through the hydrated polymer matrix. Furthermore, the diffusion exponent obtained from the Korsmeyer–Peppas model (n=0.365) provides additional mechanistic insight. Since the calculated exponent remained below 0.45, the release mechanism can be classified as Fickian diffusion, indicating that SCP transport occurs primarily through concentration-driven molecular diffusion rather than polymer relaxation or hydrogel erosion.

**Figure 7:**
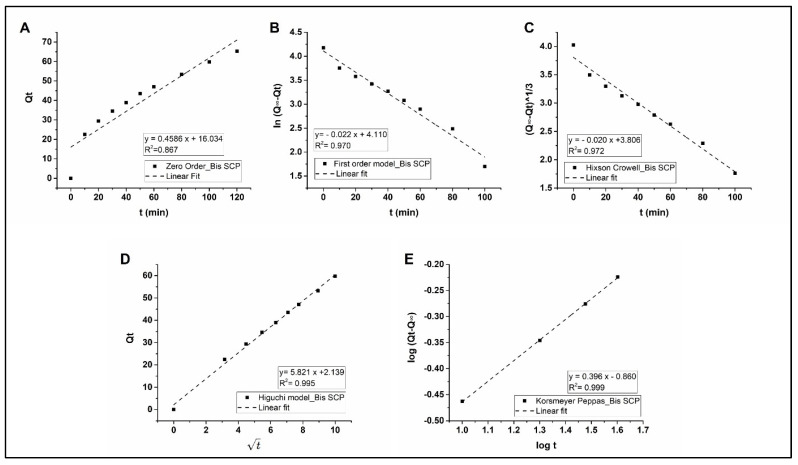
Linear fitting of the experimental release data for Bis SCP released from PVA–borax hydrogels using different kinetic models: (**A**) zero-order, (**B**) first-order, (**C**) Hixson–Crowell, (**D**) Higuchi, and (**E**) Korsmeyer–Peppas.

A similar kinetic behavior was observed for the Bis SCP/PVA hydrogel (Figure 7). The zero-order model again exhibited the weakest fitting performance (Figure 7A, R^2^ = 0.867), while the first-order (Figure 7B, R^2^ = 0.970) and Hixson–Crowell (Figure 7C, R^2^ = 0.972) models produced improved correlations, suggesting that concentration-dependent release and gradual changes in diffusion geometry contribute to the overall release process. As observed for SCP/PVA, the highest correlation coefficients were obtained using the Higuchi (Figure 7D, R^2^ = 0.995) and Korsmeyer–Peppas (Figure 7E, R^2^ = 0.999) models. The diffusion exponent calculated from the Korsmeyer–Peppas equation (n=0.396) remained below the critical limit for anomalous transport, confirming that Bis SCP release also follows a Fickian diffusion mechanism.

Although both systems exhibited diffusion-controlled release, the slightly higher diffusion exponent and larger Higuchi release constant obtained for Bis SCP (k_H_ = 5.821) compared with SCP (k_H_ = 4.001) indicate somewhat enhanced molecular transport within the Bis SCP-containing network. To further quantify transport behavior, apparent diffusion coefficients were estimated using the Higuchi model and hydrogel geometry. The calculated diffusion coefficients were on the order of 10^−11^–10^−10^ m^2^.s ^1^, confirming slow, diffusion-controlled transport through the hydrated matrix (Table 3). Bis SCP/PVA exhibited a higher diffusion coefficient than SCP/PVA, indicating that incorporation of the bis-spiro derivative facilitates molecular transport without altering the fundamental Fickian release mechanism. It should be emphasized that the assignment of a Fickian diffusion mechanism in this study rests on the fit quality (R^2^) and diffusion exponent (*n*) obtained from the Korsmeyer–Peppas and Higuchi models applied to the same cumulative-release dataset. While these parameters are the conventional and widely accepted basis for mechanistic classification in the pharmaceutical release literature, they are internally derived from the release curve itself and do not constitute an independent physical measurement of diffusivity. Accordingly, the diffusion-controlled transport mechanism proposed here should be regarded as the interpretation most consistent with the kinetic modeling results and is corroborated qualitatively by the structural (SEM), swelling, and rheological trends discussed below.

The combined SEM, FTIR, swelling, rheological, and release results reveal a clear structure–property relationship governed by phosphazene architecture. SCP-containing hydrogels exhibited higher swelling ratios and greater storage modulus values than their Bis SCP counterparts, suggesting stronger interactions between the SCP molecules and the PVA–borax network. The presence of free nitrogens on the phosphazene ring and P–Cl groups in SCP may enhance intermolecular interactions with the PVA–borax network, as supported by the FTIR red shift observed upon integration of SCP into the PVA matrix. In contrast, the bulkier Bis SCP derivative produced a more heterogeneous network morphology, as evidenced by SEM analysis, with wider and more extensively interconnected crack domains (Figure 2G–I). Although Bis SCP-containing hydrogels displayed lower overall equilibrium swelling ratios than SCP/PVA, this structural heterogeneity may create localized free volume and short-range diffusion pathways within the matrix’s hydrated regions, independent of bulk water-uptake capacity, thereby facilitating faster phosphazene transport. This interpretation is consistent with the higher Higuchi release constant (k_H_ = 5.821 vs. 4.001) and apparent diffusion coefficient (1.57 × 10^−10^ vs. 7.4 × 10^−11^ m^2^s^−1^) obtained for Bis SCP/PVA. Collectively, these findings demonstrate that the degree of spiro substitution influences network organization, water accessibility, and molecular transport, thereby governing the overall performance of phosphazene-loaded PVA–borax hydrogels.

## 3. Materials and Methods

### 3.1. Materials

Potassium carbonate (K_2_CO_3_, ≥99%), acetone (anhydrous, ≥99.5%), 2,2′-biphenol (≥98%), hexachlorocyclotriphosphazene (HCCP, ≥98%), poly(vinyl alcohol) (PVA, Mᴡ = 30,000 g mol^−1^, 87–89% hydrolyzed), and sodium tetraborate (borax, ≥99.0%) were purchased from Sigma-Aldrich (St. Louis, MO, USA) and used as received without further purification. Absolute ethanol (99.8%, Merck, Darmstadt, Germany) and deionized water (resistivity ≥18.2 MΩ cm, Millipore Milli-Q, Darmstadt, Germany) were utilized. Phosphate-buffered saline (PBS, 10 mM) with a pH of 7.4 was prepared from sodium dihydrogen phosphate and disodium hydrogen phosphate obtained from Sigma-Aldrich (≥99%), and its pH was verified with a calibrated pH meter (Mettler Toledo FiveEasy, Columbus, OH, USA) prior to use. Dimethyl sulfoxide (DMSO, ≥99.9%, Sigma-Aldrich) was used as a co-solvent in the release medium.

### 3.2. Synthesis of SCP and Bis SCP

SCP and Bis SCP were prepared as previously described [23]. Briefly, HCCP and 2,2′-biphenol were reacted in the presence of K_2_CO_3_ as a base in anhydrous acetone. Experimental details are provided in the Supporting Information (Figures S1 and S2). Mono-spiro (SCP) and bis-spiro (Bis SCP) products were isolated by column chromatography and their identities confirmed by FTIR, ^1^H-NMR, ^13^C-NMR, and ^31^P-NMR, as reported in Supporting Information (Figures S2–S10).

### 3.3. Preparation of PVA–Borax Hydrogels

#### 3.3.1. Pristine PVA–Borax Hydrogel

PVA (5.0 g) was dissolved in 50 mL of deionized water under magnetic stirring at 70 °C for 3 h to obtain a 10% (*w*/*v*) PVA stock solution. A separate 4% (*w*/*v*) borax stock solution was made by dissolving borax (2 g) in 50 mL of deionized water at room temperature (25 °C) while stirring for 30 min. The pristine PVA–borax hydrogel was prepared by adding 10 mL of the borax solution to 10 mL of the PVA solution under magnetic stirring at 200 rpm for 15 min to form the gel network. The resulting mixture was transferred to Petri dishes (diameter 5 cm) and subjected to three freeze–thaw cycles, each consisting of 12 h at −20 °C followed by 12 h at 25 °C. The obtained hydrogels were stored at 4 °C prior to characterization.

#### 3.3.2. SCP/PVA–Borax Hydrogel

SCP-loaded PVA–borax hydrogels were prepared by first crosslinking PVA and SCP with borax, followed by a freeze–thaw gelation process [33]. Either 50 mg (5 wt% loading) or 100 mg (10 wt% loading) of SCP was dissolved in 1 mL of absolute ethanol under sonication (ultrasonic bath, 40 kHz, 10 min) until completely dissolved. The SCP–ethanol solution was added dropwise to 10 mL of the 10% (*w*/*v*) PVA aqueous stock solution, and the mixture was sonicated for a further 10 min to achieve a homogeneous dispersion. Subsequently, 9 mL of 4% (*w*/*v*) borax solution was added to the PVA–SCP dispersion under magnetic stirring at 200 rpm to initiate crosslinking; stirring was continued for 15 min. Note: the borax volume in this formulation (9 mL) differs from that used for pristine PVA (10 mL) to maintain a constant total volume of 20 mL, accounting for the 1 mL of ethanol added with the phosphazene. The resulting gel was cast into Petri dishes and subjected to three freeze–thaw cycles (freezing at −20 °C for 12 h; thawing at 25 °C for 12 h). The obtained hydrogels were washed three times with deionized water (10 mL per wash) to remove residual ethanol and unreacted components, verified by UV–Vis absorbance of the wash fractions, and stored at 4 °C until use. As SCP and Bis SCP are water-insoluble, aqueous washing was performed solely to remove residual ethanol and unreacted impurities. UV–Vis analysis of the washing fractions confirmed no detectable phosphazene loss, indicating that nominal and actual retained loadings are equivalent. Therefore, phosphazene contents of the resulting formulations were 5 wt% and 10 wt% SCP, respectively.

#### 3.3.3. Bis SCP/PVA–Borax Hydrogels

Bis SCP-loaded hydrogels were prepared by an identical procedure to that described for SCP–PVA hydrogels. Bis SCP (50 mg for 5 wt%, or 100 mg for 10 wt%) was dissolved in 1 mL of absolute ethanol, incorporated into 10 mL of PVA stock solution by sonication, crosslinked with 9 mL of borax solution, and processed through three freeze–thaw cycles and washing steps as described above. All other formulation parameters were kept constant to ensure direct comparability between SCP- and Bis SCP-containing systems.

### 3.4. Swelling Measurements

The swelling behavior of the hydrogels was evaluated gravimetrically at 25 °C in deionized water. Hydrogel disks (diameter 1.5 cm, thickness 3 mm) were cut from the prepared slabs, gently blotted with filter paper to remove excess water, and their initial mass (W_0_) was recorded on an analytical balance (readability 0.1 mg). Samples were then immersed in 20 mL of deionized water at 25 °C. At predetermined time intervals (0, 15, 30, 45, 60, 75, 90, 105, 120, and 150 s after immersion), each sample was removed, blotted lightly with filter paper to remove excess surface water, and immediately weighed (W_t_). The swelling ratio (SR, %) was calculated as shown in Equation (1) [34].

SR (%) = [(W_t_ − W_0_)/W_0_] × 100 (1)

Each measurement was performed in three independent replicates (*n* = 3), and the average values were used to generate the swelling curve.

### 3.5. In Vitro Release Study and Release Kinetic Analysis

The in vitro release behavior of SCP and Bis SCP from their respective PVA–borax hydrogels was investigated using UV–Vis spectroscopy. Prior to release experiments, calibration curves for SCP and Bis SCP were constructed by preparing a series of standard solutions in PBS:DMSO (1:1, pH = 7.4) at concentrations ranging from 2 to 100 μg mL^−1^. Absorbance was measured at the maximum absorption wavelength (λ_max_) of each compound using a UV–Vis spectrophotometer (Cary 100 Bio, Varian, Palo Alto, CA, USA). Calibration curves were linear over the investigated concentration range (R^2^ > 0.999 for both SCP and Bis SCP), and the resulting equations were used to convert absorbance to phosphazene concentration throughout the study. Release experiments were conducted using the 10 wt% SCP/PVA and 10 wt% Bis SCP/PVA hydrogels. The 10 wt% formulations were selected to ensure sufficient phosphazene loading for reliable quantification of release profiles and kinetic modeling, while allowing a direct comparison of the effect of phosphazene structure on release behavior.

For release experiments, 25 mg of the hydrogel disks (10 wt% SCP or Bis SCP, diameter 1.5 cm, thickness 3 mm) were immersed in 50 mL of PBS: DMSO (1:1, pH = 7.4) at 25 °C under static conditions. The PBS: DMSO medium was selected to ensure adequate solubility of the hydrophobic phosphazene derivatives while maintaining a physiologically relevant pH. At predetermined time intervals (0, 10, 20, 30, 40, 50, 60, 80, 100, and 120 min), Aliquots of 1 mL were periodically withdrawn and replaced with the same volume of fresh medium to ensure sink conditions were maintained. The absorbance of the collected samples was measured at the corresponding λ_max_ using UV–Vis spectroscopy. The calibration equation was used to determine the cumulative amount released at time *t* (Q_t_, μg), accounting for sample replacement volume correction. Release studies were conducted in triplicate (n = 3), and the mean release profile was used to fit the kinetic model. Release kinetics were evaluated by fitting the cumulative release data to five mathematical models: zero-order, first-order, Higuchi, Hixson–Crowell, and Korsmeyer–Peppas [35,36] (Table 4).

Linear regression was used for model fitting, and the coefficient of determination (R^2^) was used to assess the goodness of fit. The transport mechanism was identified based on the Korsmeyer–Peppas diffusion exponent (n), where *n* ≤ 0.45 corresponds to Fickian diffusion, 0.45 < *n* < 0.89 indicates anomalous transport, and *n* ≥ 0.89 signifies Case II transport governed by polymer relaxation in slab-shaped systems. The apparent diffusion coefficient $\left(D\right)$ was estimated using the early-time Fickian diffusion approximation for slab geometry given in Equation (2) [37].

(2) $$\frac{Q_{t}}{Q_{\infty }}=4{\left(\frac{Dt}{\pi L^{2}}\right)}^{1/2}$$

where *Q_t_* is the cumulative amount released at time *t*, *Q_∞_* is the total releasable amount at equilibrium, *D* is the apparent diffusion coefficient (m^2^.s^−1^), and *L* is the half-thickness of the hydrogel slab. Disk-shaped hydrogels with a diameter of 1.5 cm and a thickness of 3 mm were used; accordingly, *L* was taken as 1.5 × 10^−3^ m. The value of *D* was obtained from the slope of *Q_t_*/*Q_∞_* versus t^1/2^ over the linear region (*Q_t_*/*Q_∞_* ≤ 0.60).

### 3.6. Characterization

The structures of the Bis SCP and SCP compounds were characterized by ^1^H-, ^13^C-, and ^31^P- nuclear magnetic resonance (NMR) spectroscopy using a Bruker DPX–400 spectrometer (Karlsruhe, Germany). CDCl_3_ served as the solvent, while tetramethylsilane (TMS) was employed as the internal reference. Chemical shifts (δ) are expressed in parts per million (ppm), and coupling constants (J) are reported in hertz (Hz). The surface morphology of the hydrogels was investigated by scanning electron microscopy (SEM), and their elemental composition was analyzed using energy-dispersive X-ray spectroscopy (EDS) with a Carl Zeiss Microscopy GmbH, Sigma 300 VP instrument (Oberkochen, Germany) Fourier-transform infrared (FTIR) spectra were acquired on a Bruker Tensor II spectrometer over the wavenumber range of 4000–400 cm^−1^, using 16 scans at a spectral resolution of 4 cm^−1^. Background spectra were collected immediately before each sample measurement. The thermal properties of the hydrogels (5–8 mg) were investigated using a simultaneous thermogravimetric and differential scanning calorimetric analyzer (SDT Q600, TA Instruments, New Castle, DE, USA). Thermogravimetric measurements were conducted under a nitrogen atmosphere from ambient temperature to 1000 °C at a heating rate of 10 °C min^−1^. The viscoelastic behavior of the hydrogels was evaluated by oscillatory rheology using an Anton Paar GmbH, MCR 302 rheometer (Graz, Austria) fitted with a 25 mm parallel-plate geometry. The gap between the parallel plates was adjusted to 1.0 mm prior to testing. To establish the linear viscoelastic region (LVR) for each hydrogel formulation, amplitude sweep tests were first performed at a constant angular frequency of 10 rad s^−1^ across a strain range of 0.01–10%, followed by frequency sweep measurements. Frequency sweep measurements were subsequently conducted within the confirmed LVR (strain amplitude 0.1%) over an angular frequency range of 0.1–100 rad s^−1^ at 25 °C. The storage modulus (G′), loss modulus (G″), and damping factor (tanδ = G″/G′) were recorded as functions of angular frequency. All measurements were performed in triplicate (*n* = 3).

## 4. Conclusions

This study demonstrates that the molecular architecture of spiro-dioxy-biphenyl cyclotriphosphazene derivatives plays a decisive role in regulating the structure and performance of PVA–borax hydrogels. While SCP promoted network expansion and mechanical reinforcement, Bis SCP generated a more heterogeneous structure with enhanced molecular transport and thermal resistance. Release kinetic analysis revealed that both phosphazene derivatives were released predominantly through a Fickian diffusion mechanism governed by the hydrated hydrogel network. The observed correlations between morphology, swelling behavior, viscoelastic properties, thermal stability, and release performance highlight the importance of phosphazene’s structure in tailoring hydrogel functionality. Overall, controlling the degree of spiro substitution offers a versatile strategy for designing phosphazene-functionalized hydrogels with tunable properties for controlled release and advanced functional material applications.

## Figures and Tables

**Table 1:** Characteristic FTIR absorption bands of the phosphazene ring.

Samples	*ν(P=N)* cm^−1^	*ν(P-O-C)* cm^−1^
Bis SCP	1163, 1179, 1229	951
Bis SCP/PVA/borax	1093, 1169, 1230	948
SCP	1174, 1211, 1228	953
SCP/PVA/borax	995, 1073, 1117	942

**Table 2:** Kinetic parameters obtained from different release models for SCP/PVA and Bis-SCP/PVA hydrogels.

	SCP/PVA Hydrogel		Bis SCP/PVA Hydrogel	
Model	Linear Equation	R^2^	Linear Equation	R^2^
Zero-order	*Q_t_* = 0.316*t* + 12.082	0.859	*Q_t_* = 0.4586*t* + 16.034	0.867
First-order	*ln*(*Q*_∞_ − *Q_t_*) = −0.022*t* + 3.736	0.970	*ln*(*Q*_∞_ − *Q_t_*) = −0.022*t* + 4.110	0.970
Hixson–Crowell	(*Q*_∞_ − *Q_t_*)^1/3^ = −0.017*t* + 3.361	0.970	(*Q*_∞_ − *Q_t_*)^1/3^ = −0.020*t* + 3.806	0.972
Higuchi	*Q_t_* = 4.001*t*^1/2^ + 2.504	0.994	*Q_t_* = 5.821*t*^1/2^ + 2.139	0.995
Korsmeyer–Peppas	*log*(*Q_t_*/*Q*_∞_) = 0.365*logt* − 0.805	0.999	*log*(*Q_t_*/*Q*_∞_) = 0.396*logt* − 0.860	0.999

**Table 3:** Diffusion exponent (n) and transport mechanism.

Sample	Higuchi Constant, *k_H_*	*n*	Apparent Diffusion Coefficient, *D*(m^2^/s)	Mechanism
SCP/PVA	4.001	0.365	7.4 × 10^−11^	Fickian diffusion
Bis SCP/PVA	5.821	0.396	1.57 × 10^−10^	Fickian diffusion

**Table 4:** Mathematical equations of the kinetic models used to evaluate the release behavior of SCP and Bis SCP from PVA–borax hydrogels.

Model	Mathematical Equation	Linearized Form	Plot
Zero-order	(*Q_t_* = *Q*_0_ + *k*_0_*t*)	(*Q_t_* = *k*_0_*t* + *Q*_0_)	(*Q_t_*) vs. (*t*)
First-order	*In*(*Q*_∞_ − *Q_t_*) = *InQ*_∞_ − *k*_1_ · *t*	*In*(*Q*_∞_ − *Q_t_*) = − *k*_1_ · *t* + *InQ*_∞_	*In*(*Q*_∞_ − *Q_t_*) vs. *t*
Hixson–Crowell	$Q_{\infty }^{1/3}$ − (*Q*_∞_ − *Q_t_*)^1/3^ = *k_HC_* · *t*	(*Q*_∞_ − *Q_t_*)^1/3^ = −*k_HC_* · *t* + $Q_{\infty }^{1/3}$	(*Q*_∞_ − *Q_t_*)^1/3^ vs. *t*
Higuchi	*Q_t_* = *k_H_* · *t* ^1/2^	*Q_t_* = *k_H_* · *t* ^1/2^	*Q_t_*
Korsmeyer–Peppas	*Q_t_*/*Q*_∞_ = *k_KP_* · *t^n^*	*log(Q_t_/Q_∞_) = log k_KP_ + n · logt*	*log*(*Q_t_*/*Q*_∞_) vs. *logt*

## Data Availability

The data is available upon request from the corresponding author.

## Supplementary Materials

The following supporting information can be downloaded at: https://www.mdpi.com/article/10.3390/molecules31142463/s1, Figure S1: Synthesis route of the SCP; Figure S2: Synthesis route of the Bis SCP; Figure S3: FTIR of the SCP in KBr disk; Figure S4: ^1^H-NMR spectrum of SCP (400 MHz, CDCl_3_, ppm); Figure S5:^13^C-NMR spectrum of SCP (100 MHz, CDCl_3_, ppm); Figure S6: ^31^P-NMR spectrum of SCP (162 MHz, CDCl_3_, ppm); Figure S7:FTIR spectrum of Bis SCP in KBr disk; Figure S8: ^1^H-NMR spectrum of Bis SCP (400 MHz, CDCl_3_, ppm); Figure S9: ^13^C-NMR spectrum of Bis SCP (100 MHz, CDCl_3_, ppm); Figure S10: ^31^P-NMR spectrum of Bis SCP (162 MHz, CDCl_3_, ppm); Figure S11: SEM-EDS analysis of PVA-borax hydrogel; Figure S12: SEM-EDS analysis of 10% SCP/PVA hydrogel; Figure S13: SEM-EDS analysis of 10% Bis SCP/PVA hydrogel; Figure S14: Calibration curves of SCP (A) and Bis SCP (B); Figure S15: Time-dependent release of SCP (A) and Bis SCP (B) from PVA hydrogel.
