# Supplementary material for: PVA–Borax Hydrogels Loaded with Mono- and Bis-Spiro-Dioxy-Biphenyl-Cyclotriphosphazenes: Fabrication, Physicochemical Properties, and Release Kinetics
Source: Molecules. 2026 Jul 14;31(14):2463. doi: 10.3390/molecules31142463 (PMC13414431; doi:10.3390/molecules31142463)
Supplement: Supplementary file 1 [file molecules-31-02463-s001.zip › molecules-4393478-supplementary.pdf]

1. Synthesis and Characterization of 2,2,4,4-Tetrachloro-6,6-[Spiro(2′,2″-dioxy-1′,1″-biphenylyl)]cyclotriphosphazene (SCP)

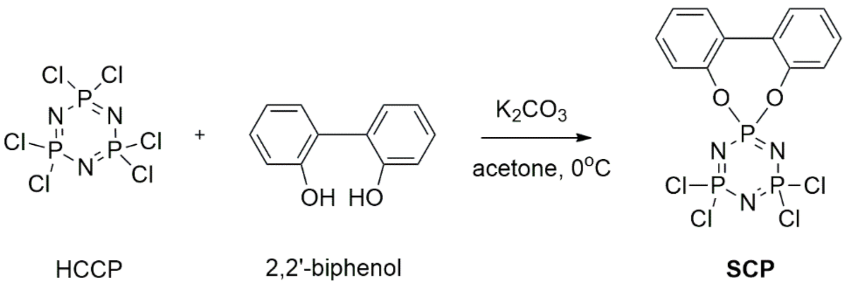

Figure S1. Synthesis route of the SCP.

Under an argon atmosphere, K_2CO_3 (147.7 mmol) was placed in a round-bottom flask, followed by the addition of acetone (50 mL) and 2,2’-biphenol (29.4 mmol). The reaction mixture was cooled to 0°C using an ice bath and stirred magnetically for 5–10 minutes. Hexachlorocyclotriphosphazene (HCCP) (29.4 mmol) was then added to initiate the reaction. Stirring was continued at room temperature for 15 minutes. After completion of the reaction, the acetone was removed using a rotary evaporator. The residue remaining in the flask was extracted with dichloromethane (3 × 50 mL). Evaporation of the dichloromethane on a rotary evaporator afforded a white solid. Recrystallization of the obtained white solid from a dichloromethane/petroleum ether (1:2) mixture gave compound SCP (12.2 g) in 90% yield. The molecular weight of compound SCP was determined to be 460.94 g/mol. Elemental analysis (%) calculated for $C_{12}H_8O_2Cl_4N_3P_3$: C, 31.3; H, 1.7; N, 9.1. Found: C, 31.5; H, 1.6; N, 8.8.

2. Synthesis and Characterization of 2,2-Dichloro-4,4,6,6-bis[spiro(2′,2″-dioxy-1′,1″-biphenylyl)cyclotriphosphazene] (Bis SCP)

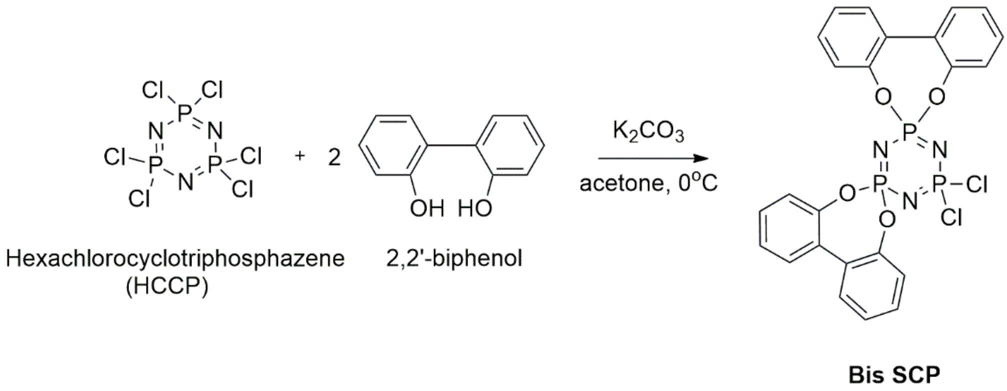

Figure S2. Synthesis route of the Bis SCP.

Under an argon atmosphere, K_2CO_3 (145 mmol) was placed in a 250 mL round-bottom flask, followed by the addition of acetone (50 mL) and 2,2′-biphenol (57.5 mmol). The reaction mixture was cooled to 0°C using an ice bath and stirred magnetically for 5–10 min. Subsequently, HCCP (29.4 mmol) was added to initiate the reaction. The reaction was allowed to proceed for 2 h at room temperature under an argon atmosphere. Upon completion, the reaction mixture was filtered under vacuum, and the solid residue remaining on the filter paper was washed with acetone. After washing, the material retained on the filter paper was extracted with dichloromethane (5 × 50 mL). Removal of the dichloromethane under reduced pressure using a rotary evaporator afforded a white solid product. The BIF compound was recrystallized from acetone. A total of 15.2 g of the Bis SCP compound was obtained, corresponding to an 87% yield. The molecular weight of the obtained Bis SCP compound was determined to be 574.23 g mol^{-1}. Elemental analysis (%) calculated for $C_{24}H_{16}O_4Cl_2N_3P_3$: C, 50.2; H, 2.8; N, 7.3. Found: C, 49.6; H, 2.6; N, 7.1.

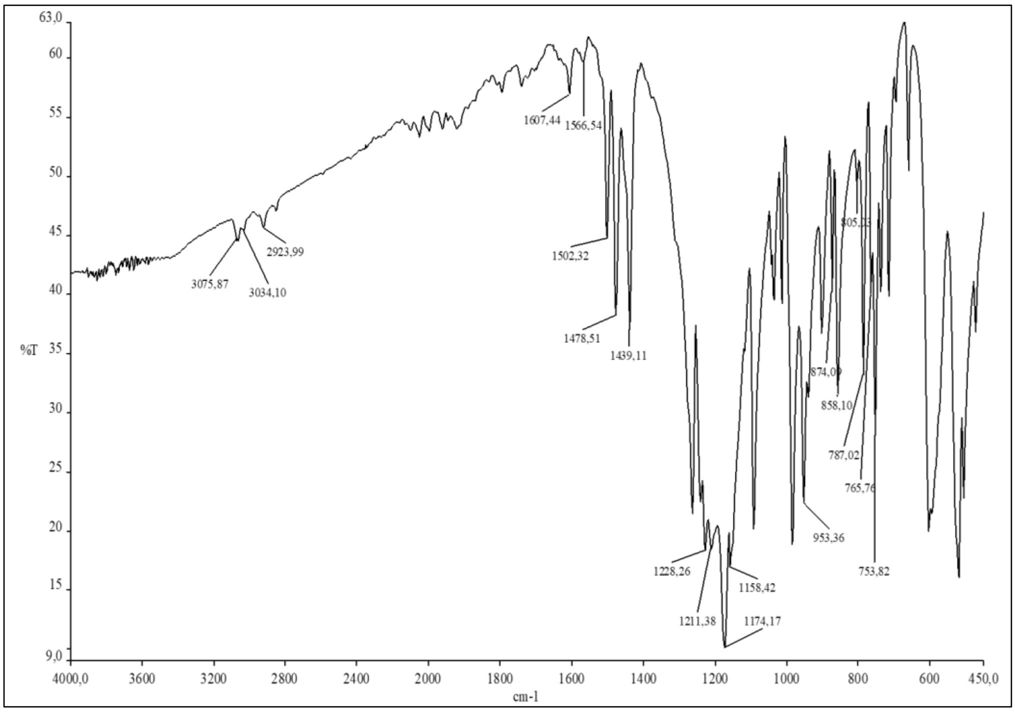

Figure S3. FTIR of the SCP in KBr disk.

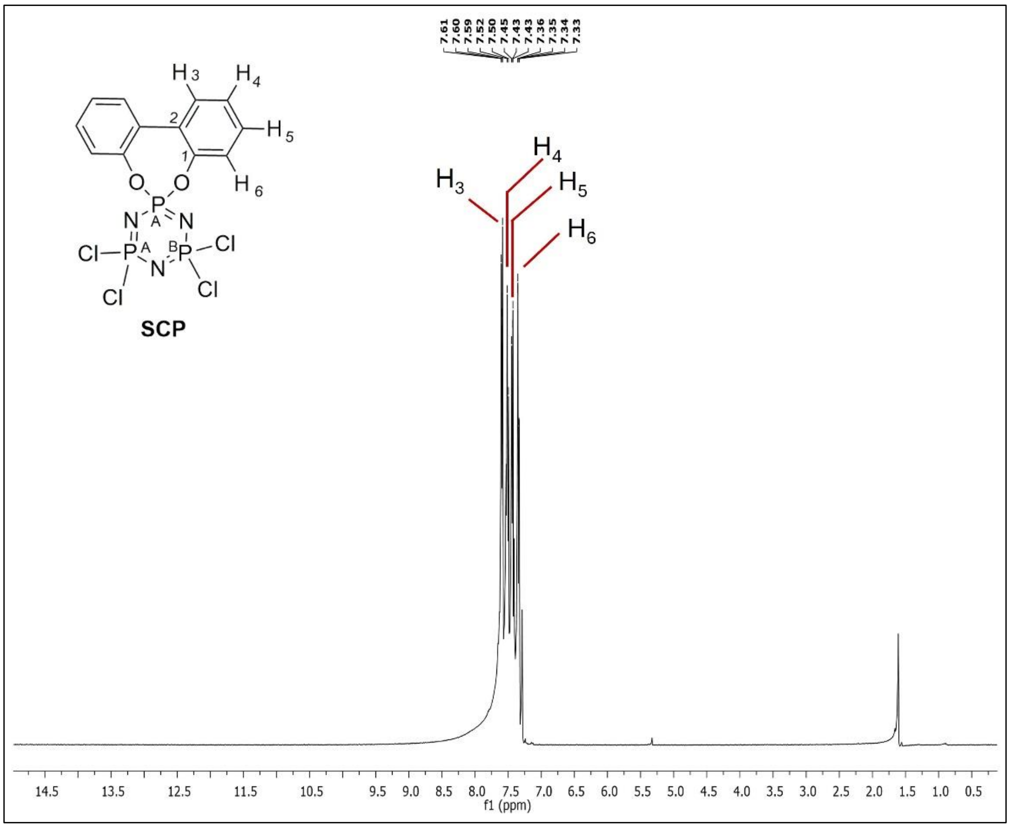

Figure S4. ^{1}H-NMR spectrum of SCP (400 MHz, $CDCl_3$, ppm).

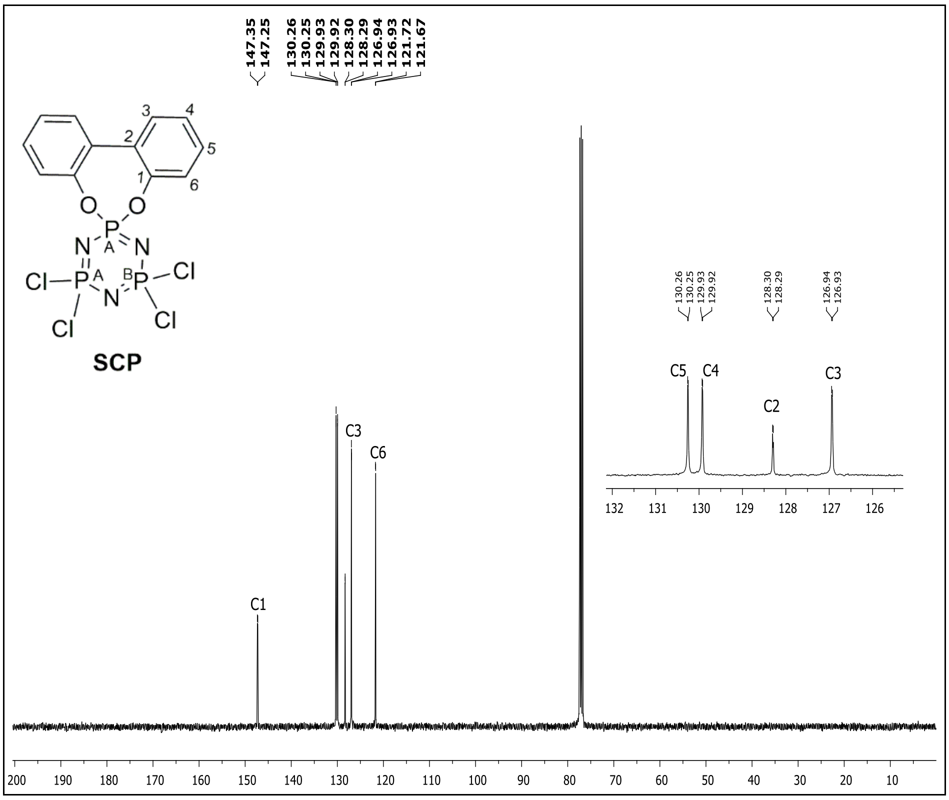

Figure S5. ^{13}C-NMR spectrum of SCP (100 MHz, $CDCl_3$, ppm).

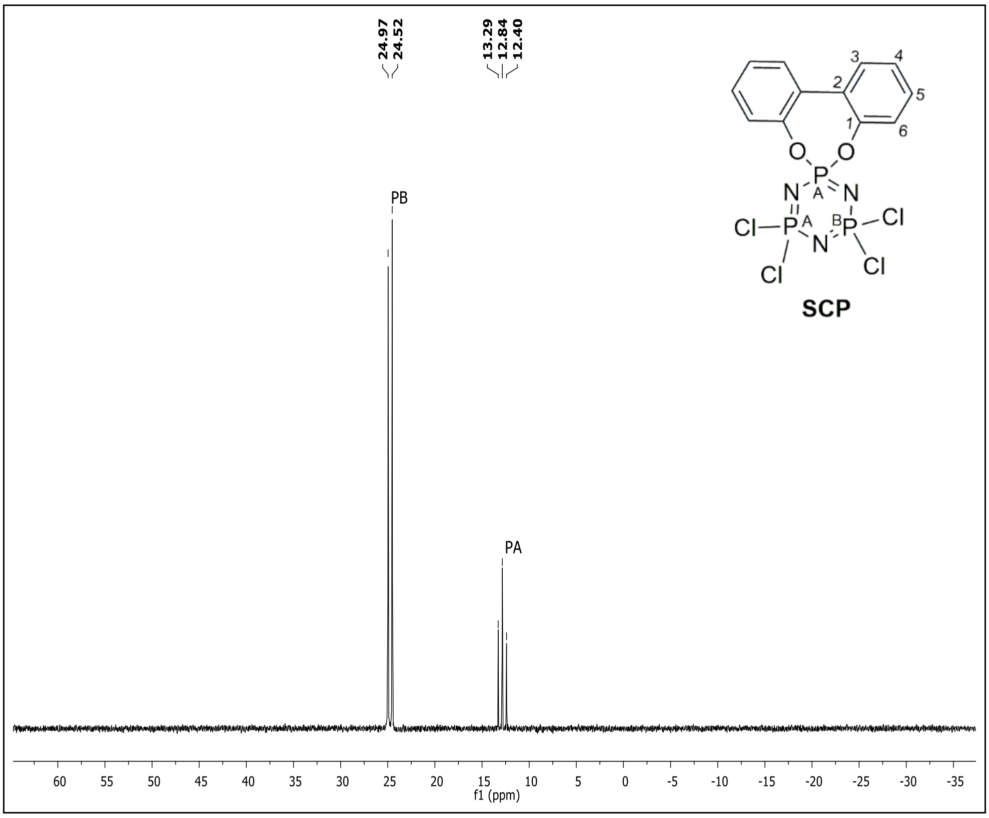

Figure S6. ^{31}P-NMR spectrum of SCP (162 MHz, $CDCl_3$, ppm).

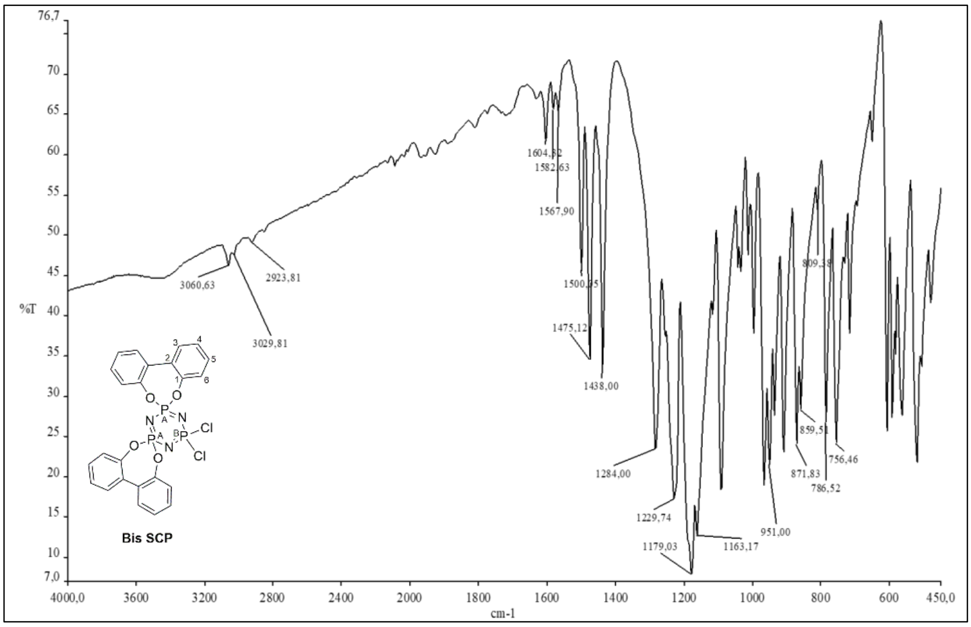

Figure S7. FTIR spectrum of Bis SCP in KBr disk.

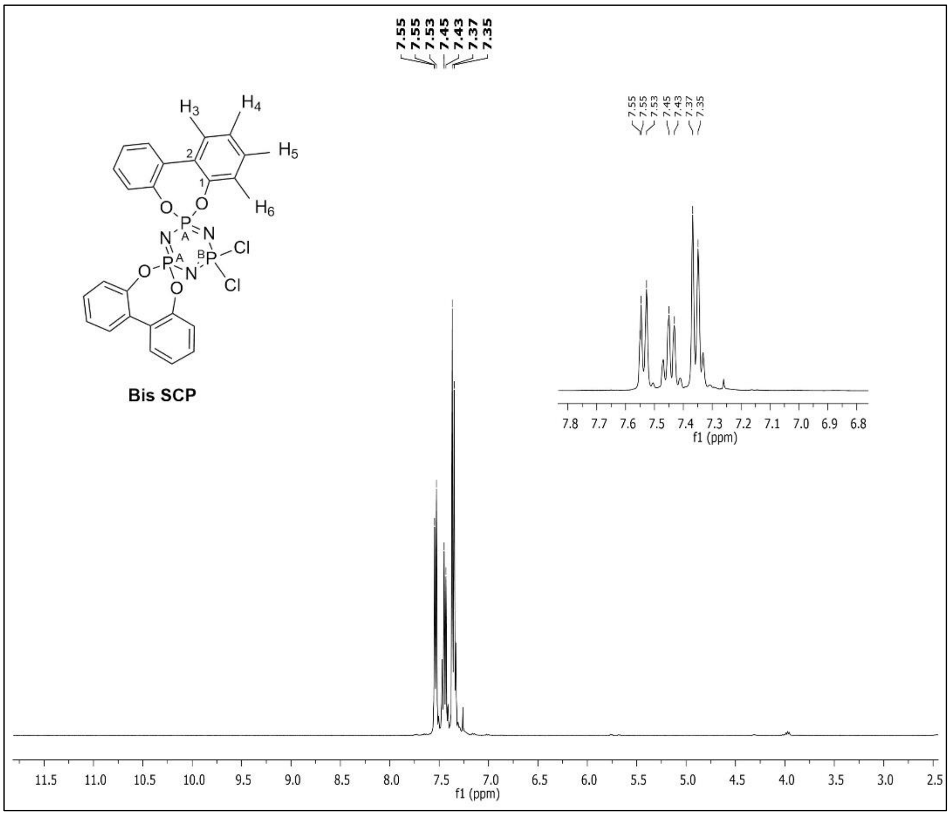

Figure S8. ^{1}H-NMR spectrum of Bis SCP (400 MHz, $CDCl_3$, ppm).

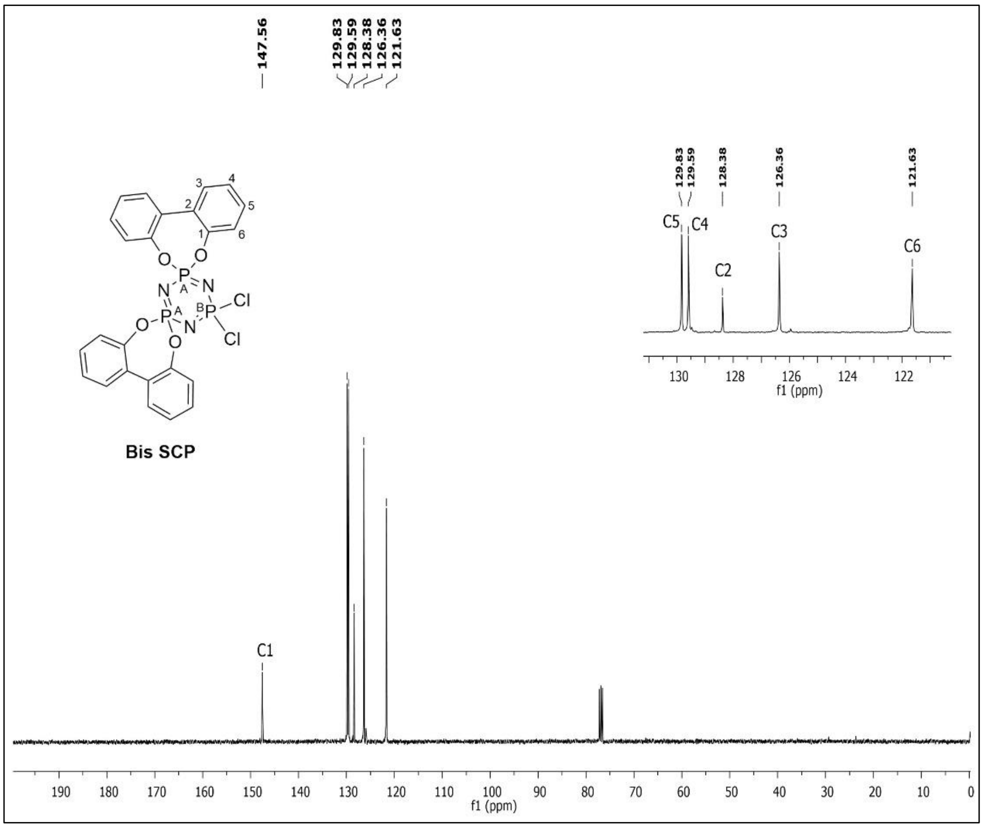

Figure S9. ^{13}C-NMR spectrum of Bis SCP (100 MHz, $CDCl_3$, ppm).

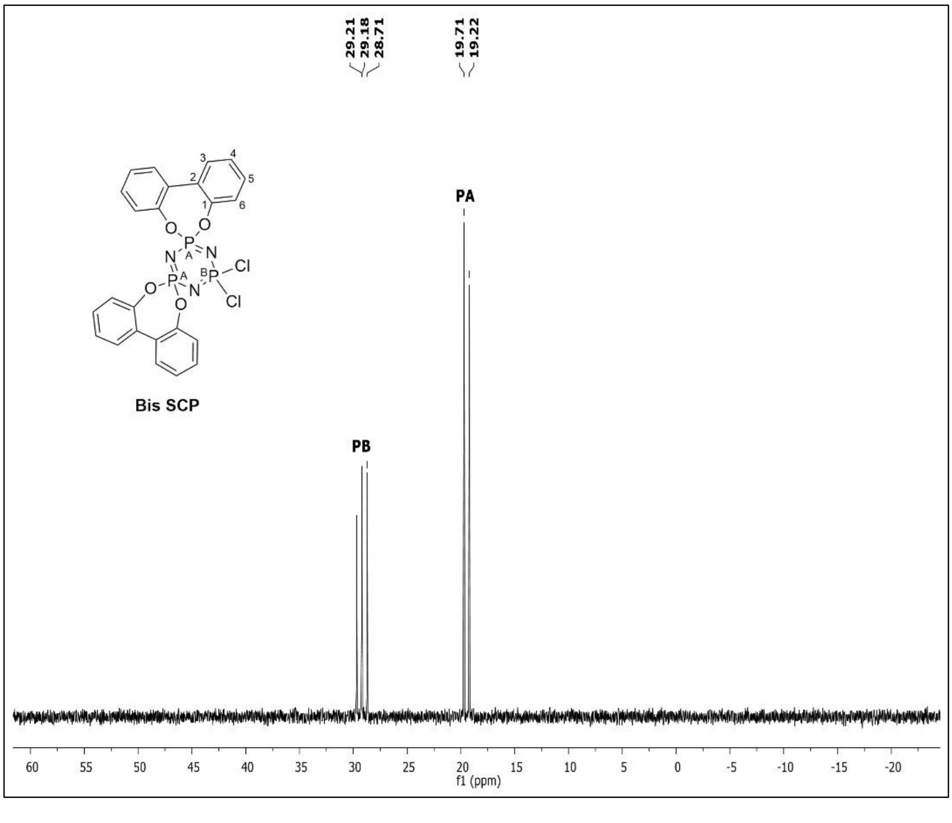

Figure S10. ^{31}P-NMR spectrum of Bis SCP (162 MHz, $CDCl_3$, ppm).

SEM-EDS Analysis

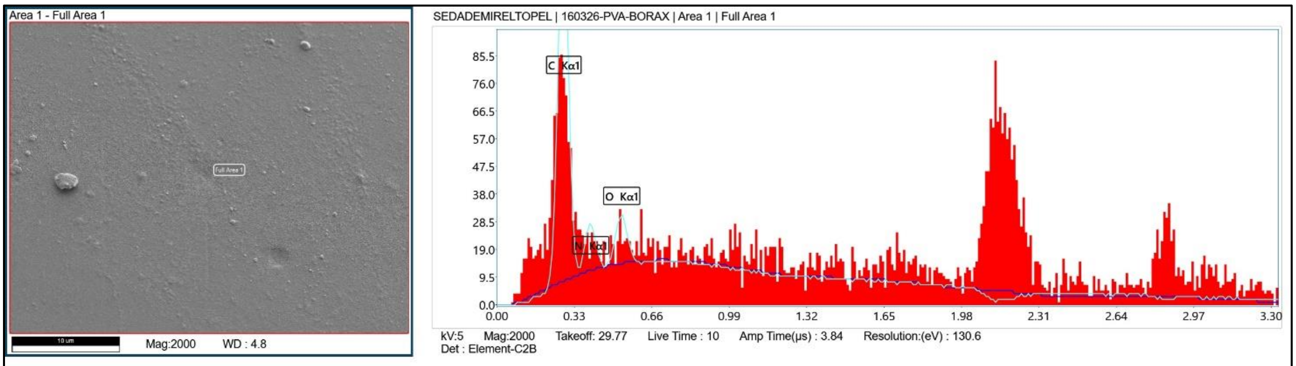

Element	Weight %	Atomic %	Error %	Net Int.	R	A	F
C K	68.22	72.57	10.71	72.07	0.9509	0.8326	1.0000
N K	18.16	16.56	38.33	9.70	0.9562	0.4542	1.0000
O K	13.61	10.87	41.68	10.77	0.9604	0.5867	1.0000

Figure S11. SEM-EDS analysis of PVA-borax hydrogel.

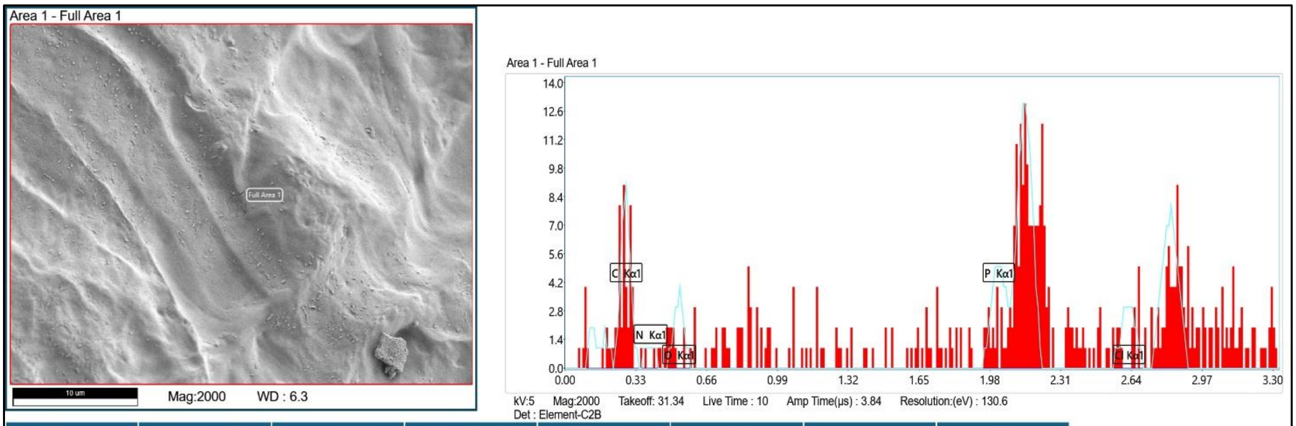

Element	Weight %	Atomic %	Error %	Net Int.	R	A	F
C K	27.13	47.77	32.47	5.16	0.9044	0.2683	1.0000
N K	3.28	4.95	99.99	0.82	0.9130	0.3786	1.0000
O K	4.59	6.06	73.20	1.99	0.9200	0.5732	1.0000
P K	28.41	19.40	15.08	5.28	0.9621	0.9851	1.0171
Cl K	36.59	21.83	20.99	3.32	0.9744	0.9808	1.0050

Figure S12. SEM-EDS analysis of 10% SCP/PVA hydrogel.

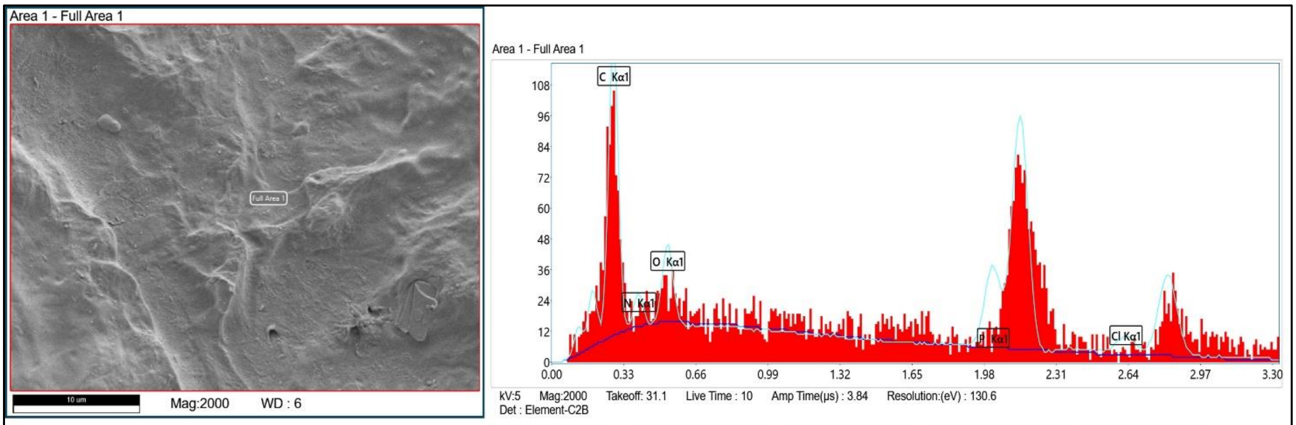

Element	Weight %	Atomic %	Error %	Net Int.	R	A	F
C K	44.69	62.85	15.22	68.72	0.9236	0.3990	1.0000
N K	5.75	6.93	52.44	8.39	0.9310	0.4083	1.0000
O K	8.00	8.45	28.07	19.51	0.9369	0.5942	1.0000
P K	28.78	15.70	10.25	28.74	0.9717	0.9864	1.0086
Cl K	12.77	6.08	22.08	6.27	0.9812	0.9821	1.0062

Figure S13. SEM-EDS analysis of 10% Bis SCP/PVA hydrogel.

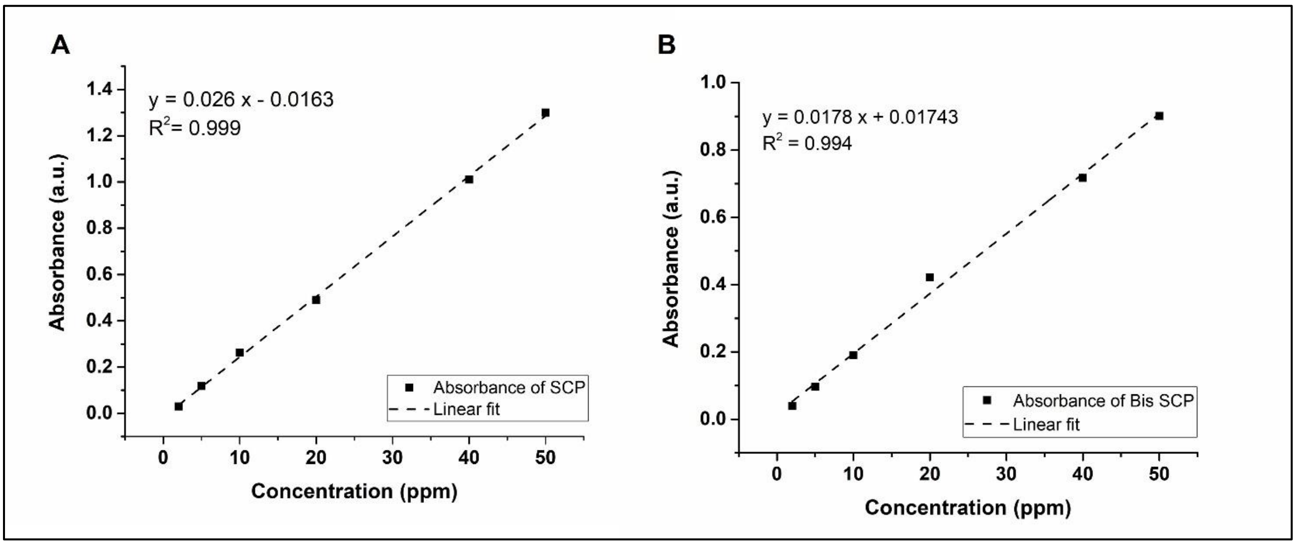

Figure S14. Calibration curves of SCP (A) and Bis SCP (B).

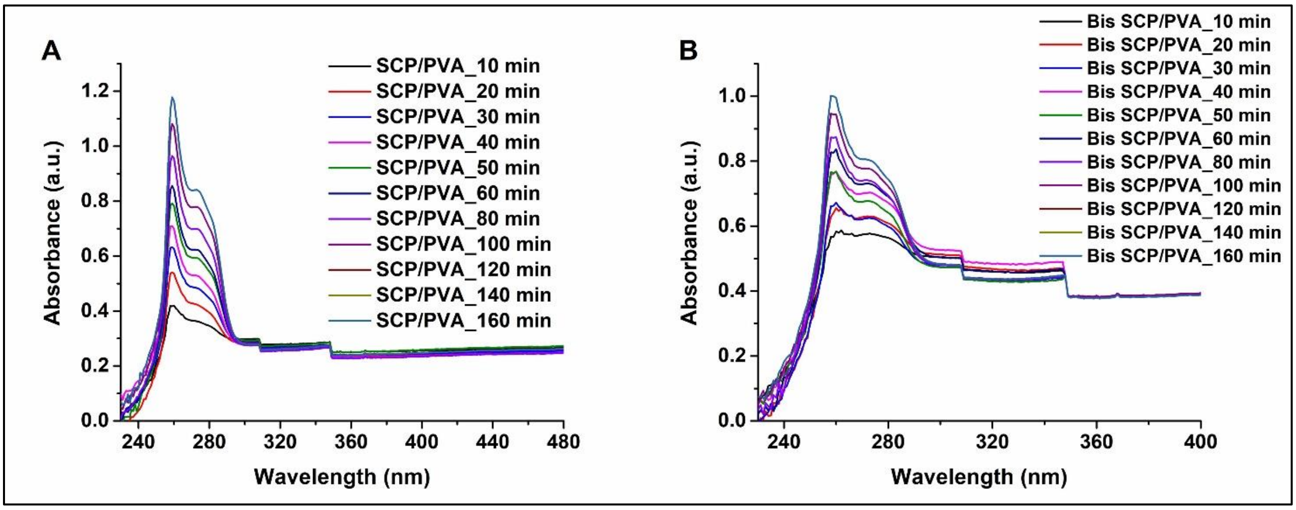

Figure S15. Time-dependent release of SCP (A) and Bis SCP (B) from PVA hydrogel.
